# Assessment of the intensive phase ‘Shakti Divas’ initiative to combatting anemia in Rajasthan, India

**DOI:** 10.1371/journal.pone.0319520

**Published:** 2025-03-13

**Authors:** Nitin Kumar Joshi, Pankaj Bhardwaj, Akhil Dhanesh Goel, Tanisha Garg, Yogesh Kumar Jain, Manoj Kumar Gupta, Prem Singh, Jitendra Kumar Soni

**Affiliations:** 1 School of Public Health, All India Institute of Medical Sciences, Jodhpur, Rajasthan, India; 2 Department of Community Medicine and Family Medicine, All India Institute of Medical Sciences, Jodhpur, Rajasthan, India; 3 National Health Mission, Government of Rajasthan, Rajasthan, India; Faculty of Medicine, Parul Institute of Public Health, Parul University, INDIA

## Abstract

**Introduction:**

Anemia is a global health concern, affecting over 2 billion people worldwide. Rajasthan state of India launched an intensive phase initiative to combat anemia comprehensively.

**Objectives:**

To assess the intensive phase anemia control “Shakti Divas” initiative of Rajasthan government through process and outcome valuation, while understanding the challenges and barriers in the implementation.

**Methods:**

A state-wide mixed methods cross-sectional survey was conducted. Process evaluation was done through data collection in seven identified districts of Rajasthan. This included surveys in Anganwadi centers and government schools, and home visits for out-of-school children, pregnant and lactating women not coming to Anganwadi centres. For outcome evaluation, IFA coverage data was obtained from government web portals one-month before and six-months after the launch of initiative. 38 in-depth interviews were conducted to assess the challenges and barriers.

**Results:**

Process evaluation - cross-sectional surveys conducted in 1100 Anganwadi centers serving 68,651 beneficiaries, and 1240 government schools serving 1,30,114 students, and home visits to survey 29,960 children and 18,632 pregnant women. 843 Anganwadi centers (76.6%), benefitting 68,651 people and 916 schools (73.8%) with 97,247 beneficiaries, were actively engaged in the initiative. Outcome evaluation - an overall increase in IFA coverage amongst beneficiary groups as well individual districts seen, with maximum increase amongst adolescent girls category (68.6%). Challenges were enlisted as knowledge, budgetary, supply, reporting, compliance, resource, coverage and monitoring constrains.

**Conclusion:**

The paper discussed the reach and challenges of an intensified initiative to combat anemia. Similar intensified and targeted strategies may serve as the key to achieve the goal of anemia reduction thus creating a healthier future for population with similar socio-demographics in LMICs.

## Introduction

Anemia is a global health concern affecting over 2 billion people worldwide. The World Health Organization (WHO) estimates a high prevalence of anemia, with rates of 40% among children aged 6 to 59 months, 37% among pregnant women, and 30% among reproductive-age women (15-49 years) [1,2]. This condition, is characterized by a lack of healthy red blood cells or insufficient hemoglobin and is particularly prevalent in developing countries including India [2].

India, as a developing nation, has a significant burden of anemia, posing a considerable public health challenge. Iron deficiency anemia is the most common form and is often treatable through dietary modifications and iron supplementation [3,4]. The prevalence of anemia being the highest among children aged 6-59 months poses a considerable threat to the upcoming younger generation [5,6]. Anemia can lead to cognitive development issues in children, affecting concentration and work performance, as well as hindering physical growth and overall fitness [7].

Anemia significantly contributes to morbidity, resulting in a loss of 19.7 million disability-adjusted life years [8,9]. Recognizing the gravity of the situation, India has implemented various anemia control programs over the years, evolving strategies, target groups, interventions, and diagnostic methods to enhance program effectiveness. The most recent initiative, “Anemia Mukt Bharat” (AMB), launched in 2018, and employs a comprehensive “6x6x6 strategy”. It focuses on improving Iron and Folic Acid (IFA) coverage among six beneficiary groups, strengthening health systems through six institutional measures, and implementing six programmatic initiatives to reduce anemia prevalence [10].

These beneficiary groups encompass children aged 6-59 months, children aged 5-9 years, adolescents aged 10-19 years (both boys and girls), reproductive-age women 15-49 years, pregnant women, and lactating mothers. The multifaceted AMB initiative combines various interventions, including IFA supplementation, deworming, behavior change communication, digital testing for anemia, and fortified foods, to tackle anemia comprehensively [3,10].

Nevertheless, despite such initiatives, the prevalence of anemia has continued to rise in India, as indicated by the National Family Health Survey-5 (NFHS-5) data. This concerning trend signified the need for intensified efforts to combat anemia effectively. In response, the government of Rajasthan, launched the “Shakti Divas” initiative in June 2022 to help achieve the targets of Anemia Mukt Rajasthan.

This study was planned to assessment the Shakti Divas Initiative of the Government of Rajasthan through process evaluation and outcome evaluation, and understand the challenges and barriers in implementing the initiative.

## Methodology

A state-wide mixed methods cross-sectional survey was carried out in the selected districts in the state of Rajasthan for the evaluation of implementation and operations of the Shakti Divas initiative. A pool of relatively low-performing districts with respect to Iron Folic Acid (IFA) coverage was developed, and seven districts were randomly selected for the surveys. For process evaluation, data collection was conducted for all the activities that were included under the initiative. Surveys were conducted in Anganwadi centers and government schools within the selected districts from May 15, 2023 to July 14, 2023. Furthermore, home visits were organized to reach out to out-of-school children, pregnant women, and lactating women who were not coming to Anganwadi centers. Written informed consent was obtained from the study participants, and in case of minors, the same was obtained from their parents.

While, for outcome evaluation, data regarding the IFA coverage before and after the implementation of the initiative were obtained for different beneficiary groups from Web portals (Anemia Mukt Rajasthan portal), Government documents (NHM office, Education department, Women and Child Development department), and available literature (NFHS reports). The data was analyzed using Microsoft Excel and percentage change in IFA coverage one month before (May 2022), and six months after (December 2022) the launch of the initiative was calculated. Improvements in the IFA coverage was represented amongst different beneficiary groups. The gathered data was obtained in anonymised format with no access to information that could potentially identify the individual participants.

Assessment of the challenges and barriers in the implementation of the initiative was done through the in-depth interviews conducted at the implementation sites, namely Anganwadi centers, government schools, subcenters, health and wellness centers, Primary Health Centers (PHCs), Community Health Centers (CHCs), and government medical institutions. Study participants were Shakti Divas beneficiaries and key stakeholders who were chosen through the convenience sampling method. Interviews were taken from ANM, ASHA, Students in government schools, Government school teachers, doctors, and government officials from the health and education department, and continued till the data saturation. A total of thirty-eight interviews were conducted using a semi- structured questionnaire. Interviews were transcribed and translated and thematic analysis was done to understand the challenges and barriers in the initiative.

Ethics statement: Written informed consent was obtained from the study participants, and in case of minors, the same was obtained from their parents. The ethical approval for the study was taken from the Institutional Ethics Committee of All India Institute of Medical Sciences, Jodhpur (Rajasthan, India) vide certificate reference number: AIIMS/IEC/2023/5676.

## Results

It was observed that the Shakti Divas initiative was planned to decrease the prevalence of anemia by screening the beneficiaries based on physical symptoms, hemoglobin testing, providing Standard of Care to screened patients, and referral of severe cases to higher centers. The program implemented in joint collaborations between Health Department, Department of School Education and the Women and Child Development, and the initiative is mandated to be organised on every Tuesday of the month wherein, “divas” translates to “day” in the local vernacular.

### Process evaluation

**Fig 1:**
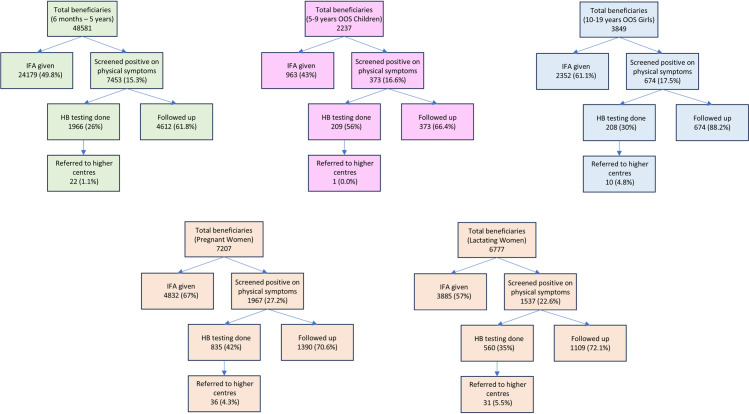
Assessment of activities at Anganwadi Centres of seven districts of Rajasthan. Process evaluation of activities at Anganwadi centres for different beneficiary subsets – (top left to top right) **(A)** Children aged 6 months to 5 years, **(B)** Out of school children aged 5 years to 9 years, **(C)** Out of school girls aged 10 years to 19 years, (bottom left to bottom right) **(D)** Pregnant women, **(E)** Lactating women.

During the assessment, surveys were conducted in total of 1100 Anganwadi centers and 1240 government schools. Additionally, surveys were also conducted through home visits. Out of a total of 1100 Anganwadi centers, a significant majority of 843 centers (76.6%) actively organized “Shakti Divas” events. A comprehensive evaluation of the 68,651 beneficiaries from Anganwadi centers is described in Fig 1.

Line listing of the beneficiaries was successfully done at 73.6% of the Anganwadi centers. Specific, line listings for children aged 6 months to 5 years was available at 95% of the centers, while the line listing for pregnant women was available at 91% of the centers. Lists for lactating women was found at 88% of the centers, for out-of-the-school girls aged 10-19 years at 59% centers, and for out-of-the-school children aged 5-9 years at 32% of the centers. A total of 1543 meetings were organized to educate beneficiaries, with participation from over 6000 women and over 500 adolescent girls. Information, Education and Communication (IEC) materials were displayed at only 150 Anganwadi centers, and primarily focused on promoting locally available iron-rich food. A total of 622 social behavior change activities were conducted during this period, out of which, 189 incorporated videos, and 164 included engaging educational games for effectively conveying messages and promote behavioral change (Data in S1 Text).

**Fig 2:**
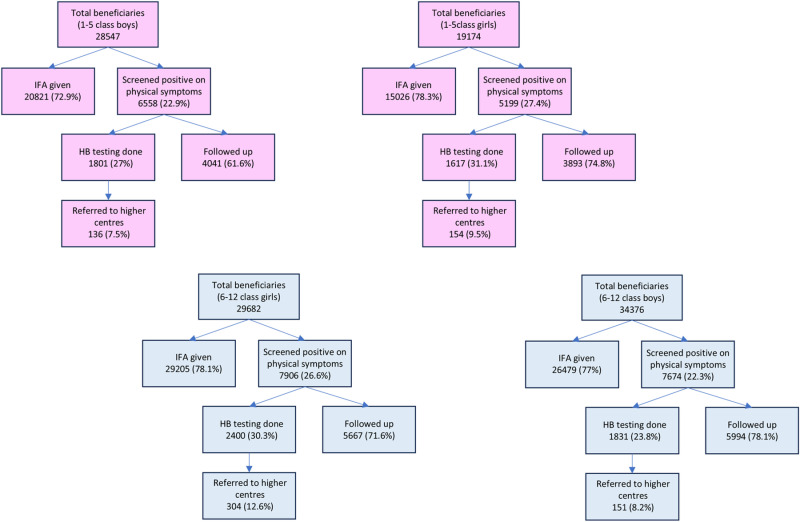
Assessment of activities at government schools of Rajasthan during the intensive phase Shakti Divas initiative. Process evaluation of activities at government schools for different beneficiary subsets – (top left to top right) **(A)** Class 1 to Class 5 boys, **(B)** Class 1 to Class 5 girls, (bottom left to bottom right) **(C)** Class 6 to Class 12 girls, **(D)** Class 6 to Class 12 boys.

Amongst 1240 government schools, with 1,30,114 enrolled students, “Shakti Divas” was organized in 916 (73.8%) schools. The assessment in schools was done based on the indicators mentioned in Fig 2. Shakti Divas monitors were designated in all the schools, while line listing of the beneficiaries was done in 54% of the schools out of which 69% had listings for 1-5 class boys and girls and 39% had listings available for 6-12 class boys and girls.

A total of 945 meetings were conducted in the government schools in the seven surveyed districts in the duration of six months, which were attended by 58,248 boys and 33,755 girls, and 5244 parents of the children. Only 124 activities for promoting social and behavioral change were carried out using games and 99 activities were conducted using videos. In total 566 schools representing 61.7% of schools had IEC material and 130 of them had educational materials that included information about locally available iron-rich foods.

In addition to this, surveys were conducted through home visits to evaluate the status of iron and folic acid (IFA) supplementation across various demographic segments and age groups. Among children aged 6-59 months in all seven districts, a total of 29,960 children were surveyed revealing that 50.4% had IFA bottles available at home and 48% had consumed IFA syrup, with an average of 8 doses in a month. Additionally, 33% of the caregivers demonstrated knowledge of the correct administration of the IFA syrup and proper storage of the IFA bottles. For the children between 5 to 9 years who were out of school, 2535 children were surveyed, with 50.8% having received IFA pink tablets, and 45.6% consuming the provided IFA tablets. Among out-of-school adolescent girls aged 10 to 19 years, 8335 surveys were conducted, showing that 59.8% of them had access to IFA blue tablets, and 56.2% had effectively consumed the IFA blue tablets as recommended.

Out of 18,632 surveyed pregnant women, 68.1% had received IFA red tablets, and 65% responded having properly consuming the provided IFA red tablets. Similarly, among the lactating women, 18,498 surveys were carried out, indicating that 58.4% received IFA red tablets, and 54.7% properly consumed the IFA red tablets.

### Outcome evaluation

For the outcome evaluation of the initiative, reports and documents on IFA coverage in the state of Rajasthan were analysed. It was observed that the AMB index, which is defined as the simple average IFA supplementation coverage across all beneficiary groups, improved from 52.9 in quarter three to 58.5 in quarter four. With the improvement of the AMB index, rank of Rajasthan improved from 21 in quarter three to 11 in quarter four.

Monthly reports of IFA coverage among different beneficiary groups were analyzed for the month of May 2022 (which is one month prior to the launch of initiative) and for the month of December 2022 (six months after the implementation). IFA coverage for the respective months were analyzed in different age groups and the percentage change in IFA coverage was assessed (Table 1, Table A in S2 Text and Fig A in S2 Text).

**Table 1:** Iron folic acid coverage among different beneficiary groups.

	AMR program indicators	Coverage (%) in May 2022	Coverage (%) in December 2022	Percentage improvement
1	Children (6-59 Month) Given 8 or more doses Of IFA Syrup	35.8%	48.8%	13.0%
2	Children (5-9 Years) consume at least 4 IFA Pink tablets	7.3%	40.3%	33.0%
3	Adolescent (10-19 Years) Boys consume at least 4 IFA tablets	21.9%	89.9%	68.0%
4	Adolescent (10-19 Years) Girls consume at least 4 IFA tablets	22.7%	91.3%	68.6%
5	Pregnant women given 180 IFA red tablets during pregnancy	92.1%	95%	2.9%

There was overall progress in IFA coverage across different groups. For children (6-59 months) receiving 8 + doses of IFA syrup, coverage improved by 13%, while for children (5-9 years) consuming at least 4 IFA Pink tablets, it rose by 33%. Coverage among adolescents (10-19 years) also improved—boys’ coverage increased from 21.9% to 89.90%, and girls’ from 22.7% to 91.30% between May and December 2022. Pregnant women saw a rise from an already high 92.10% to 95%.

The highest percentage increases were in adolescents (10-19 years), with boys and girls improving by 68% and 68.6%, respectively. The lowest coverage was in children (6-59 months) and (5-9 years).

**Fig 3:**
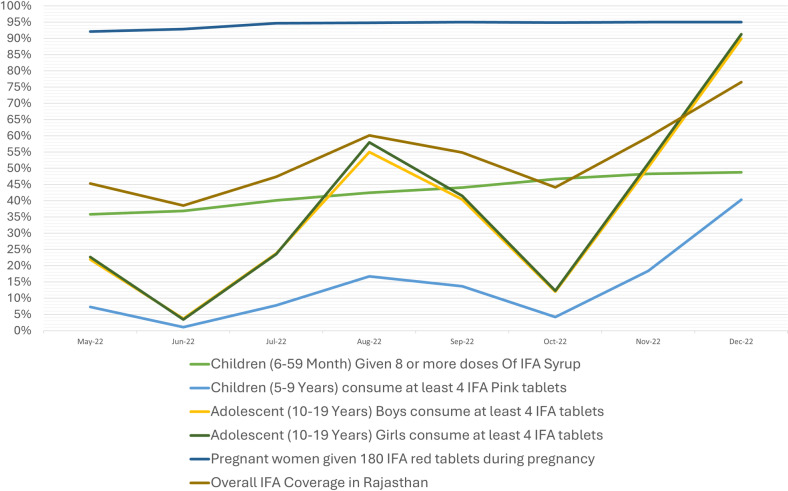
Iron folic acid (IFA) coverage amongst different beneficiary groups.

Fig 3 shows the IFA coverage among different beneficiary groups.

### IFA coverage

#### IFA Syrup – beneficiaries aged 6-59 months.

IFA Syrup is administered at Anganwadi centers, with reporting by ASHA. A steady rise in coverage was seen from May to December 2022. By January 2023, 48% of districts had coverage between 50-75%, an improvement from 50-25% in May 2022.

#### IFA pink coverage – beneficiaries aged 5-9 years.

Coverage saw fluctuations, starting below 10% in May 2022, dipping to 1.10% in June, and rising to 4.20% by October. By January 2023, 97% of districts had below 25% coverage, with only 6% improvement, mainly due to school vacations.

#### IFA blue coverage – beneficiaries aged 10-19 years, boys and girls.

IFA Blue is distributed in schools, showing a similar increasing trend for both boys and girls, with dips in June and October 2022 due to vacations. By January 2023, 42% of districts had coverage below 25% for boys (down from 67% in May), while for girls, this dropped from 64% to 45%.

#### IFA red coverage - pregnant women.

IFA Red distribution during antenatal care showed strong coverage, increasing from 92.10% in May to 95% in December 2022.

#### Overall coverage in Rajasthan.

Coverage rose from 45.28% in May 2022 to 76.52% in December 2022, with notable dips in June and October due to school vacations.

#### District-level IFA coverage.

Districts with initially low coverage improved by over 40%, while those with higher coverage saw less than 20% improvement after the initiative (District and Zone-wise coverage, monthly scores described in Tables B–H and Fig B in S2 Text).

### Assessment of challenges and barriers

Thirty-eight in-depth interviews with policymakers, policy implementers, and beneficiaries were conducted, including ASHA workers (6), ANMs (7), beneficiaries–students, pregnant and lactating women (10), medical officers (7), nodal officers (2) and officials from ICDS and education departments (6). Common challenges and barriers were identified across themes.

#### Knowledge constraints.

During interviews, stakeholders revealed a knowledge gap among ASHA, ANM, and teachers about the initiative’s full scope. Most were only aware of iron and folic acid tablet distribution, unaware of essential activities like screening, hemoglobin testing, and awareness sessions. Training for screening anemia and using digital hemoglobinometers was requested. Teachers lacked training to administer medications, affecting students’ education time.

*“We are only told to administer tablets, so we do that only.” (ASHA Worker)*

*“We only give Iron Tablets.” (ASHA Worker)*

Many stakeholders misunderstood prophylactic treatment, associating anemia with dietary issues rather than seeing the importance of preventive care.

*“According to us, only those with paleness should be given the tablets.” (ANM Staff)*

#### Budget constraints.

Stakeholders raised concerns about hemoglobinometer procurement due to budget limitations. While devices were initially available, stakeholders could not replace or repair them when faulty.

*“We don’t have an instrument for Hemoglobin testing as we have no budget.” (Medical Officer)*

*“We had hemoglobinometers, but once they became defective, we couldn’t replace them.” (Medical Officer)*

#### Supply constraints.

Stakeholders reported IFA (Iron and Folic Acid) supply shortages, despite improvements with Shakti Divas. The availability of IFA pink and blue variants remained insufficient. They proposed a direct supply to schools instead of teachers collecting it from PHCs. The weekly supply system was also deemed inadequate, with suggestions for a monthly supply.

*“We are not getting IFA blue supply.” (Medical Officer)*

*“Every Tuesday, teachers’ time gets wasted in going to PHC, sometimes without success.” (Education Office representative)*

#### Reporting constraints.

Stakeholders expressed the need for an online platform for reporting 126 weekly indicators, including automated calculations. The absence of structured data formats hindered tracking anemic patients and resolving duplicity issues.

*“Google sheet reports should be used to track anemic individuals.” (ICDS Office representative)*

*“Our teachers face problems in reporting.” (Education office representative)*

#### Compliance constraints.

Patients often discontinued medications due to side effects like nausea, impacting treatment adherence. Stakeholders suggested emphasizing dietary iron sources, especially iron-rich millets in Rajasthan. Digital media could help promote these options, and fortified foods could boost compliance.

*“Fortified food should be made available to improve compliance.” (Child Development Office representative)*

*“Emphasis should be on millet, which is rich in iron.” (Child Development Office representative)*

#### Resource constraints.

Insufficient staffing was another key issue. Teachers were burdened with medical tasks, disrupting education. The appointment of school nurses was suggested. Additionally, patients had to travel to hospitals for hemoglobin testing, leading to poor follow-up.

*“There should be a school nurse appointed. Teachers are overworked and untrained.” (Education Office representative)*

*“For hemoglobin testing, we send patients to the nearby PHC.” (Medical Officer)*

#### Monitoring constraints.

The program lacks a proper monitoring mechanism, essential for ensuring its effectiveness.

*“The program should be monitored regularly.” (ICDS Office representative)*

#### Coverage constraints.

Inadequate IFA coverage was identified being centered around PHCs, CHCs, and Anganwadi centers. The current strategy targets underprivileged groups, leaving out affluent individuals, many of whom also suffer from anemia.

*“Many students in private schools are not covered.” (ASHA Worker)*

*“Distribution should be replanned to cover the entire population.” (ASHA Worker)*

## Discussion

Anemia remains a major public health challenge, especially among women and children in our country. Iron deficiency anemia is a leading cause, and despite efforts by central and state governments through programs like the National Nutritional Anemia Control Program, WIFS, and NIPI, anemia prevalence in Rajasthan has increased. This research assessed the challenges and barriers in implementing the ‘Shakti Divas’ initiative and its effectiveness.

One challenge identified was interdepartmental coordination between the health, education, and women and child development departments. The education department reported that teachers were untrained for medical tasks, making implementation difficult. Joe et al., 2022, also found weak IFA coverage across different groups supplied by different departments [3]. Bhatia et al., 2019, emphasized the need for interdepartmental coordination to strengthen programs [2].

IFA supply issues were another key constraint. Inconsistent supply, especially of IFA pink and blue during June and October, affected coverage among adolescents. Adolescents are particularly vulnerable to anemia due to their physical and physiological demands [11]. The lead time index, which measures supply chain efficiency, was 34 weeks in Rajasthan in 2018-19, indicating inefficiency, as noted by Ahmad et al., 2023 [12]. Although supply improved with ‘Shakti Divas,’ it remains a concern.

Budget constraints for hemoglobinometers and transportation were also noted. Proper planning and adequate budget allocation are essential for efficient use of hemoglobinometers. Regular monitoring would ensure better resource utilization. Avi Saini et al., 2022, reported budget increases for AMB from FY 2019 to FY 2022, but improper planning caused budget issues [13].

There is no online platform for data reporting, making it difficult to track anemic patients. Bhatia et al., 2019, recommended a web-based monitoring system for reliable data management [2]. Despite improved IFA coverage after ‘Shakti Divas,’ compliance remains low due to gastrointestinal symptoms. Nicole U Stoffel et al., 2019, found better absorption and compliance with fractionated iron doses and morning administration [14].

‘Shakti Divas’ has raised awareness and improved IFA supplementation, with potential long-term positive effects. Similar initiatives have been shown to reduce anemia prevalence [15]. District-level analysis showed substantial improvements in IFA coverage in some areas, though variation across districts highlights the need for targeted interventions to ensure equitable coverage.

## Conclusion

The Shakti Divas initiative may be viewed is one of the successful initiatives of the Rajasthan government, with immediate output observed in form of increased IFA coverage amongst all the beneficiary groups. Due to the weekly reporting norms, activities were carried out regularly and reported every week. Short-term outcomes like increased awareness and motivation to reduce anemia were noticed among the beneficiaries with positive changes in knowledge and attitudes regarding anemia.

Whenever a new program is launched it has various challenges and barriers that needs to be addressed to bring out the maximum benefit of the program. The paper elaborately discussed few challenges such as budgetary constraints, supply issues, compliance difficulties and stakeholders coordination, that need to be taken into consideration to bring sustainable success and maximum effectiveness of the initiative and reduce anemia in Rajasthan. Continuous evaluation, adaptation, and targeted strategies could be the key to achieving the goal of anemia reduction and creating a healthier future for populations with similar demographics amongst the developing and low- and middle- income countries.

## Supporting information

S1 Text Process evaluation of anganwadi centers from selected districts of Rajasthan. IFA coverage information of various beneficiary groups with list and frequency of activities under the Shakti Divas Initiative from participating Anganwadi Centers of Rajasthan.
(PDF)

S2 Text District and Zone-wise outcome evaluation of IFA coverage in Rajasthan. District and Zone-wise coverage, monthly scores, and overall coverage in selected districts and for the state of Rajasthan.
(PDF)
