# Supplementary material for: Assessment of the intensive phase ‘Shakti Divas’ initiative to combatting anemia in Rajasthan, India
Source: PLoS One. 2025 Mar 13;20(3):e0319520. doi: 10.1371/journal.pone.0319520 (PMC11906040; doi:10.1371/journal.pone.0319520)
Supplement: S1 Text — IFA coverage information of various beneficiary groups with list and frequency of activities under the Shakti Divas Initiative from participating Anganwadi Centers of Rajasthan. (PDF) [file pone.0319520.s001.pdf]

Total Anganwadi 962

Anganwadi open 951

Aww available at 938

Sd organised on 876 Anganwadi

Not organised 61

	6m -5 years	5 years -9 years	10-19 years	pregnant women	lactating women
line listing of the beneficiaries available	830 (94.7%)	306 (34.9%)	566 (64.6%)	856 (97.7%)	842 (96.1%)
IFA insufficient	154 (17.5%)	149 (17.0%)	145 (16.5%)	133 (15.1%)	133 (1.75%)
IFA not available	48 (5.4%)	189 (21.5%)	125 (14.2%)	88 (10%)	88 (10%)
IFA sufficient	674 (76.9%)	538 (61.4%)	606 (69.1%)	655 (74.7%)	655 (74.7%)
screening present	360	57	221	424	355
screening absent	516	819	655	452	521

AWW	781 (89.1%)
Helper	621 (70.8%)
ASHA	800 (91.3%)
ANM	840 (95.8%)
RBSK Team	9 (1%)
MO	31 (3.5%)
CHO	55 (6.2%)
Department officers	4 (0.4%)
MLA	2 (0.2%)
No one present	1 (0.1%)

Awareness meetings conducted at 583 centres and not conducted at 289

Meetings for adolescent girls were conducted at 307 centers and not conducted at 567 centers

no SBCC activities	311
poster charts	462
video film	191
games	197
other material	89

IEC Flex	230
IEC posters	392
IEC leaflets	127

Charts	93
Newspaper	26
other	56
no iec	412

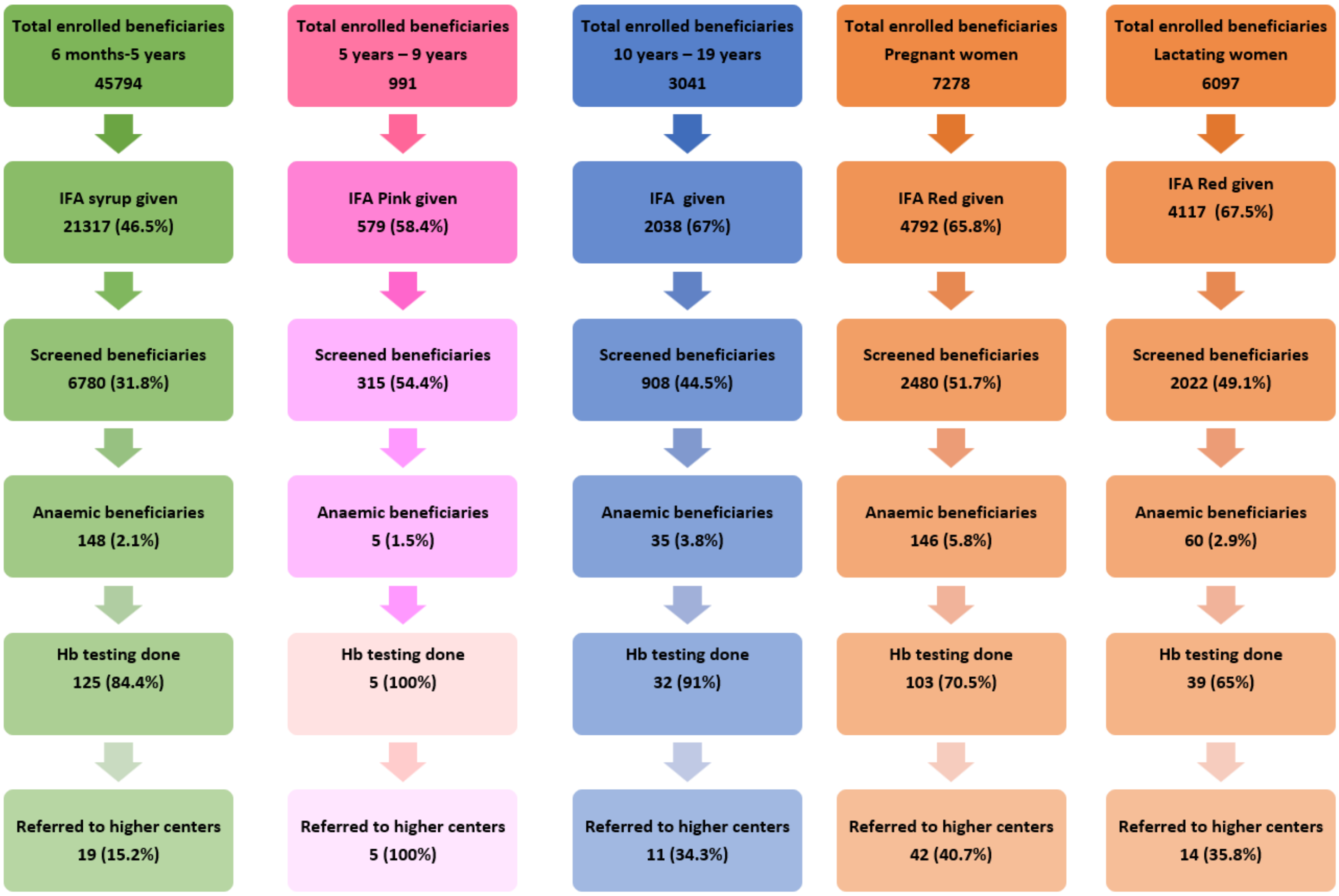

		6M-5 YEARS	5-9 YEARS	10-19 YEARS	PREGNANT WOMEN	LACTATING WOMEN
LINE LISTING OF BENEFICIRIES	YES	806	278	499	771	750
	NO	311	839	618	346	366
IFA	INSUFFICIENT	162	169	156	155	155

	NOT AVAILABLE	65	206	150	163	163
	SUFFICIENT	619	472	543	530	530
	BLANK	271	270	268	267	267(1)

Assessment of the Anganwadi centers 7 districts of Rajasthan

437 anagandwadi centers

Shakti divas organised in 327 centers

Not organised in 100 centers . 26 were not organizing due to unavailability of the supply. 25 centers didn't have asha or aww . 10 anganwadi centers were closed . no knowledge of shakti divas in 14 centers. Asha duty somewhere else in 25 centrers.

6-5 months beneficiary list is available in 318 centers , 5-9 years in 102 centers , 10-19 years in 184 centers , pw and lc women in 280 cneters

Ifa syrup was not available in 28 centers , ifa pink was not avaialbel in 93 centers ifa blue not available in 72 centers IFA red was not avaialbel in 83 centers

325 centers were having children 6-59 months , IFA syrup was administered in 298 centers , screening based on physical symptoms was done in 141 centers. Hb testing is done in 87 centers

Children 5-9 years were coming in 94 centers , ifa pink was given in 62 cneters , screening is done in 26 centers , hb testing 23 cneters

10-19 yeasr children 183 centers , ifa blue given in 133 , screening in 59, hb testing in 41 centers

154 anaganwadi cneters had IEC materials
