# Supplementary material for: Assessment of the intensive phase ‘Shakti Divas’ initiative to combatting anemia in Rajasthan, India
Source: PLoS One. 2025 Mar 13;20(3):e0319520. doi: 10.1371/journal.pone.0319520 (PMC11906040; doi:10.1371/journal.pone.0319520)
Supplement: S2 Text — District and Zone-wise coverage, monthly scores, and overall coverage in selected districts and for the state of Rajasthan. (PDF) [file pone.0319520.s002.pdf]

Table A: Overall average of Rajasthan state with respect to below 6 indicators of the program

S.No.	Program Indicators	Coverage (%) of respective KPIs for Rajasthan state in May-2022 (Average for all 33 districts)
1	Children (6-59) Month Given 8 or more doses of IFA Syrup in a month	35.80%
2	Children (5-9) Years consume at least 4 IFA Pink tablets in a month	7.30%
3	Adolescent (10-19) Years Boys consume at least 4 IFA tablets in a month	21.90%
4	Adolescent (10-19) Years Girls consume at least 4 IFA tablets in a month	22.70%
5	Adolescent (10-19)Years Out of school girls given IFA Blue tablets 4 time & above in a month	91.70%
6	Pregnant Women given 180 IFA tablets during pregnancy period.	92.10%
	Average Coverage of IFA for Rajasthan State (average of above 6 indicators)	45.30%

Figure A: Coverage percentage of KPIs for Rajasthan state in May-2022

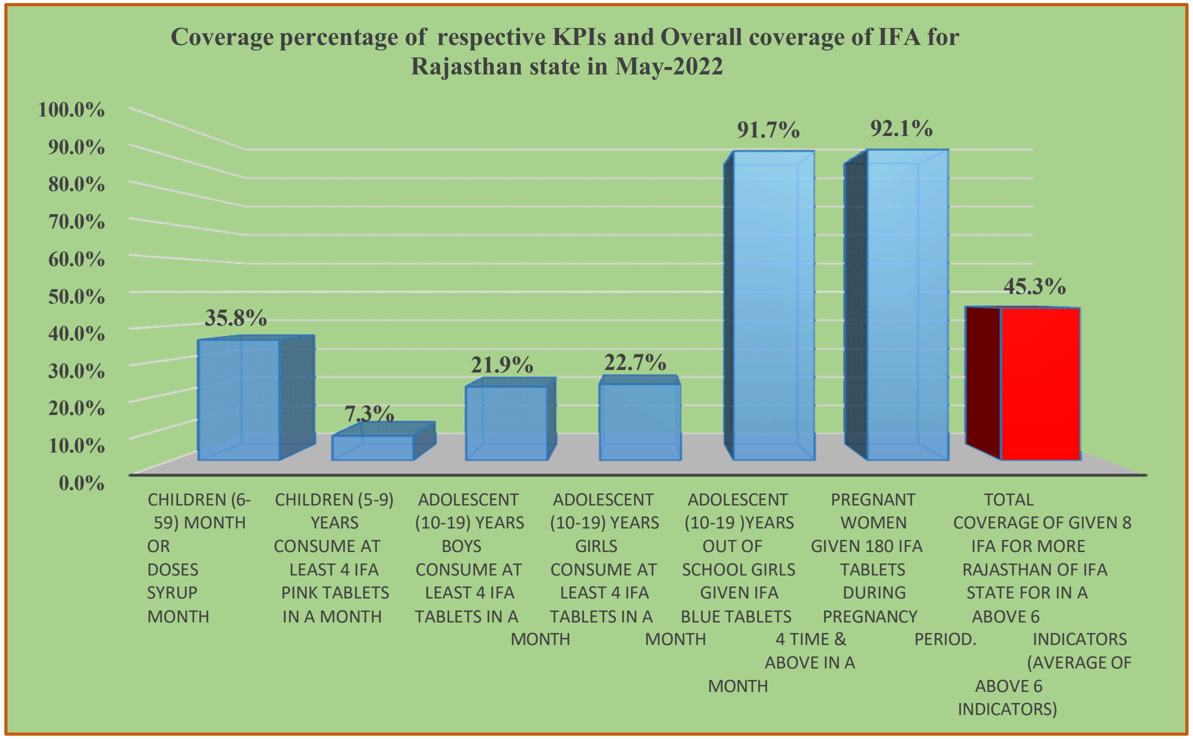

Table B: Zone-Wise Coverage for May-2022

Zone	Coverage in May-2022	Rank	Comment
Bikaner	50.00%	1	Bikaner zone has Coverage in range >= 50% and < 75%
Bharatpur	48.30%	2	
Ajmer	47.40%	3	
Udaipur	46.90%	4	
Kota	46.00%	5	All these zones have Coverage in range >= 25% and < 50%
Jodhpur	41.50%	6	
Jaipur	39.50%	7	

Graph Shows Zone Wise Overall Average Coverage (decreasing order) in May-2022

Figure B: Zone Wise Coverage for May 2022

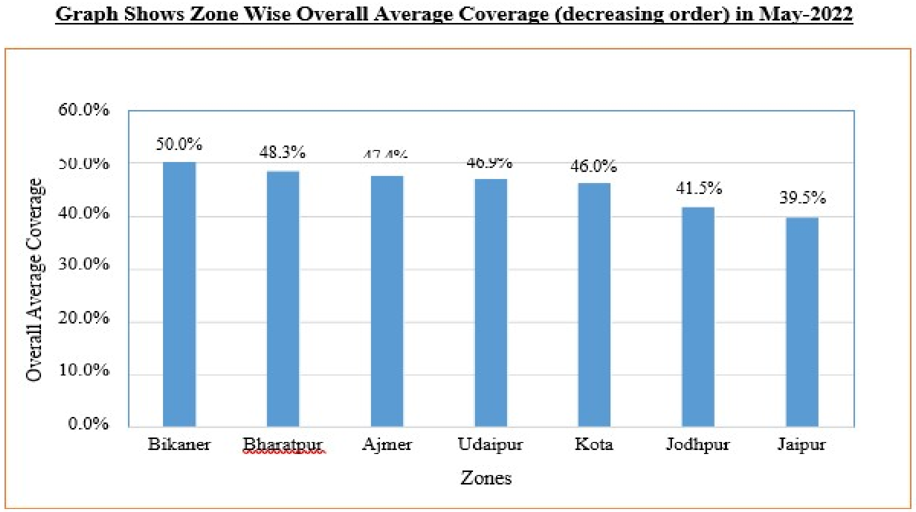

Zone	District	Cvg. in May	Comment
Ajmer	Nagaur	33.20%	
Bharatpur	Bharatpur	35.30%	
Bikaner	Churu	38.30%	Coverage in range of
Jaipur	Dausa	34.60%	>=25% and < 50%
Jaipur	Alwar	35.00%	
Jodhpur	Jaisalmer	35.90%	
Kota	Baran	42.50%	
Udaipur	Dungarpur	36.90%	

Table C: District Wise IFA coverage in May 2022

Range	No. of district in given range (Avg. Cvg.)	Top 5 districts	Coverage in May-2022	Bottom 5 districts	Coverage in May-2022
0.0% to 24.9% (Pink Color)	0, (0%)	Bhilwara	73.50%	Jaisalmer	35.90%
25.0% to 49.9% (Blue Color)	25, (76%)	Ganganagar	66.60%	Bharatpur	35.30%
50.0% to 74.9% (Yellow Color)	8, (24%)	Sawai Madhopur	63.10%	Alwar	35.00%
75.0% to 95.0% (Green Color)	0, (0%)	Chittaurgarh	58.30%	Dausa	34.60%
>95.0% (White Color)	0, (0%)	Rajsamand	57.50%	Nagaur	33.20%

Table D: District Wise Monthly Score-card FY:2022-23

as on Date: 27-June-2022

Districts	Month: May-2022	
	Overall Coverage (%)	Overall Ranking
Bhilwara	73.50%	1
Ganganagar	66.60%	2
Sawai Madhopur	63.10%	3
Chittaurgarh	58.30%	4
Rajsamand	57.50%	5
Hanumangarh	54.60%	6
Bundi	53.90%	7
Dhaulpur	51.10%	8
Banswara	48.30%	9
Jhunjhunu	48.30%	10
Jalor	48.20%	11
Tonk	46.90%	12
Kota	44.40%	13
Karauli	43.50%	14
Jhalawar	43.40%	15
Pratapgarh	42.80%	16
Baran	42.50%	17
Barmer	42.50%	18
Sirohi	42.40%	19
Sikar	42.10%	20
Jodhpur	41.50%	21
Bikaner	40.40%	22
Pali	38.50%	23
Churu	38.30%	24
Jaipur	37.50%	25
Udaipur	37.20%	26
Dungarpur	36.90%	27
Ajmer	36.00%	28
Jaisalmer	35.90%	29
Bharatpur	35.30%	30
Alwar	35.00%	31
Dausa	34.60%	32
Nagaur	33.20%	33
RAJASTHAN	45.30%	

Table E: District Wise Performance-Sheet for Last 3-Months (FY: 2022-23)

Month: May-2022

as on Date: 27-June-2022

Districts	Overall Cvg (%) in March-2022	Overall Cvg (%) in April-2022	Overall Cvg (%) in May-2022
Ajmer	27.70%	33.40%	36.00%
Alwar	30.70%	35.30%	35.00%
Banswara	26.90%	44.20%	48.30%
Baran	25.70%	37.10%	42.50%
Barmer	19.70%	46.70%	42.50%
Bharatpur	26.50%	35.00%	35.30%
Bhilwara	45.90%	41.80%	73.50%
Bikaner	25.80%	42.60%	40.40%
Bundi	36.80%	41.20%	53.90%
Chittaurgarh	49.70%	52.40%	58.30%
Churu	40.20%	37.60%	38.30%
Dausa	25.90%	33.00%	34.60%
Dhaulpur	41.30%	45.00%	51.10%
Dungarpur	26.60%	33.90%	36.90%
Ganganagar	46.10%	49.80%	66.60%
Hanumangarh	45.30%	54.10%	54.60%
Jaipur	42.50%	32.00%	37.50%
Jaisalmer	22.90%	28.20%	35.90%
Jalor	25.10%	46.20%	48.20%
Jhalawar	28.30%	38.40%	43.40%
Jhunjhunu	41.70%	48.60%	48.30%
Jodhpur	20.80%	38.90%	41.50%
Karauli	29.30%	43.70%	43.50%
Kota	36.60%	38.80%	44.40%
Nagaur	27.10%	31.90%	33.20%
Pali	43.90%	37.00%	38.50%
Pratapgarh	40.30%	38.50%	42.80%
Rajsamand	33.70%	40.00%	57.50%
Sawai Madhopur	22.90%	50.20%	63.10%
Sikar	31.80%	39.40%	42.10%
Sirohi	37.60%	29.30%	42.40%
Tonk	42.40%	41.90%	46.90%
Udaipur	41.10%	36.10%	37.20%
Rajasthan	33.60%	40.10%	45.30%

Table F: District Wise Coverage for month of May-2022 (FY:2022-23)

Month: May-2022
as on Date: 27-June-2022

S.No.	KPI / Source / Districts	Children (6-59) Month Given 8	Children (5-9) Years Consume at least 4 IFA	Adoloscent (10-19)Years Cor		Pink tablets in a / Adoloscent (1(	Pregnant Women Given 180 IFA tablets (includes 360 IFA figures)	Overall Ave	Overall Ranking (District-Wise Sorted)
	Source	(PCTS)	(Shaladarpan)	(Shaladarpan)		(PCTS)	(PCTS)		
	Districts	Coverage (%)	Coverage (%)	Boys Coverage	Girls Coverage	Girls Coverage	PW Coverage (%)		
1	Ajmer	36.40%	0.80%	2.00%	2.40%	95.00%	79.20%	36.00%	28
2	Alwar	36.00%	0.40%	3.40%	2.90%	95.00%	72.10%	35.00%	31
3	Banswara	70.40%	6.20%	10.90%	12.50%	95.00%	95.00%	48.30%	9
4	Baran	46.10%	4.70%	10.50%	10.20%	95.00%	88.50%	42.50%	17
5	Barmer	9.30%	19.90%	30.10%	32.20%	74.10%	89.30%	42.50%	18
6	Bharatpur	21.00%	0.20%	0.40%	0.40%	95.00%	95.00%	35.30%	30
7	Bhilwara	49.00%	31.00%	81.80%	89.40%	95.00%	95.00%	73.50%	1
8	Bikaner	30.20%	3.40%	9.70%	9.20%	95.00%	95.00%	40.40%	22
9	Bundi	36.40%	9.00%	37.50%	50.40%	95.00%	95.00%	53.90%	7
10	Chittaurgarh	58.10%	14.20%	48.50%	39.30%	95.00%	95.00%	58.30%	4
11	Churu	34.10%	1.20%	2.00%	2.70%	95.00%	95.00%	38.30%	24
12	Dausa	11.50%	0.90%	2.60%	2.60%	95.00%	95.00%	34.60%	32
13	Dhaulpur	41.10%	12.70%	32.10%	30.70%	95.00%	95.00%	51.10%	8
14	Dungarpur	28.80%	0.30%	1.20%	1.10%	95.00%	95.00%	36.90%	27
15	Ganganagar	56.10%	17.80%	70.10%	65.30%	95.00%	95.00%	66.60%	2
16	Hanumangarh	60.00%	6.40%	36.20%	35.20%	95.00%	95.00%	54.60%	6
17	Jaipur	15.70%	1.50%	10.30%	10.20%	95.00%	92.40%	37.50%	25
18	Jaisalmer	9.10%	8.40%	24.00%	27.30%	54.60%	91.90%	35.90%	29
19	Jalor	10.60%	15.40%	36.00%	36.90%	95.00%	95.00%	48.20%	11
20	Jhalawar	32.70%	5.10%	14.60%	18.30%	95.00%	95.00%	43.40%	15
21	Jhunjhunu	52.90%	3.80%	20.50%	22.60%	95.00%	95.00%	48.30%	10
22	Jodhpur	14.30%	5.30%	24.10%	24.50%	86.00%	94.50%	41.50%	21
23	Karauli	34.10%	9.20%	27.60%	29.20%	66.10%	95.00%	43.50%	14
24	Kota	30.00%	4.60%	20.20%	21.50%	95.00%	95.00%	44.40%	13
25	Nagaur	11.80%	0.10%	0.30%	0.30%	95.00%	91.50%	33.20%	33
26	Pali	38.50%	1.10%	0.60%	0.90%	95.00%	95.00%	38.50%	23
27	Pratapgarh	67.00%	0.00%	0.00%	0.00%	95.00%	95.00%	42.80%	16
28	Rajsamand	36.40%	20.20%	45.00%	53.40%	95.00%	95.00%	57.50%	5
29	Sawai Madhopur	27.30%	21.80%	72.70%	66.90%	95.00%	95.00%	63.10%	3
30	Sikar	39.80%	1.70%	11.40%	9.80%	95.00%	95.00%	42.10%	20
31	Sirohi	51.50%	7.10%	13.80%	14.30%	86.70%	80.80%	42.40%	19
32	Tonk	68.90%	2.10%	10.60%	9.90%	95.00%	95.00%	46.90%	12
33	Udaipur	17.50%	4.90%	14.00%	16.70%	95.00%	75.10%	37.20%	26
	Rajasthan	35.80%	7.32%	21.95%	22.70%	91.75%	92.10%	45.30%	

Table G: Rajasthan Zone & District Wise Coverage -May-2022 (FY:2022-23)

Month: May-2022
as on Date: 27-June-2022

S.No.	Zone	KPI Source Districts	Children (6-59) Month Given 8 or more ((PCTS) Coverage (%)	Children (5-9) Years Consume at least 4 IFAPink tablets in a month (Shaladarpan) Coverage (%)	Adoloscent (10-19)Years Consume at le: (Shaladarpan) Boys Coverage (%)	Girls Coverage (%)	Adoloscent (10-19)' (PCTS) Girls Coverage (%)	Pregnant Women Given 180 IFA tablets (includes 360 IFA figures) (PCTS) PW Coverage (%)	Overall Average Cov	Overall Ranking (District-Wise Sorted)
1	Ajmer	Ajmer	36.40%	0.80%	2.00%	2.40%	95.00%	79.20%	36.00%	28
2	Ajmer	Bhilwara	49.00%	31.00%	81.80%	89.40%	95.00%	95.00%	73.50%	1
3	Ajmer	Nagaur	11.80%	0.10%	0.30%	0.30%	95.00%	91.50%	33.20%	33
4	Ajmer	Tonk	68.90%	2.10%	10.60%	9.90%	95.00%	95.00%	46.90%	12
	AVG. COVERAGE OF AJMER ZONE		41.50%	8.50%	23.70%	25.50%	95.00%	90.20%	47.40%	
5	Bharatpur	Bharatpur	21.00%	0.20%	0.40%	0.40%	95.00%	95.00%	35.30%	30
6	Bharatpur	Dhaulpur	41.10%	12.70%	32.10%	30.70%	95.00%	95.00%	51.10%	8
7	Bharatpur	Karauli	34.10%	9.20%	27.60%	29.20%	66.10%	95.00%	43.50%	14
8	Bharatpur	Sawai Madhopur	27.30%	21.80%	72.70%	66.90%	95.00%	95.00%	63.10%	3
	AVG. COVERAGE OF BHARATPUR ZONE		30.90%	11.00%	33.20%	31.80%	87.80%	95.00%	48.30%	
9	Bikaner	Bikaner	30.20%	3.40%	9.70%	9.20%	95.00%	95.00%	40.40%	22
10	Bikaner	Churu	34.10%	1.20%	2.00%	2.70%	95.00%	95.00%	38.30%	24
11	Bikaner	Ganganagar	56.10%	17.80%	70.10%	65.30%	95.00%	95.00%	66.60%	2
12	Bikaner	Hanumangarh	60.00%	6.40%	36.20%	35.20%	95.00%	95.00%	54.60%	6
	AVG. COVERAGE OF BIKANER ZONE		45.10%	7.20%	29.50%	28.10%	95.00%	95.00%	50.00%	
13	Jaipur	Alwar	36.00%	0.40%	3.40%	2.90%	95.00%	72.10%	35.00%	31
14	Jaipur	Dausa	11.50%	0.90%	2.60%	2.60%	95.00%	95.00%	34.60%	32
15	Jaipur	Jaipur	15.70%	1.50%	10.30%	10.20%	95.00%	92.40%	37.50%	25
16	Jaipur	Jhunjhunu	52.90%	3.80%	20.50%	22.60%	95.00%	95.00%	48.30%	10
17	Jaipur	Sikar	39.80%	1.70%	11.40%	9.80%	95.00%	95.00%	42.10%	20
	AVG. COVERAGE OF JAIPUR ZONE		31.20%	1.70%	9.60%	9.60%	95.00%	89.90%	39.50%	
18	Jodhpur	Barmer	9.30%	19.90%	30.10%	32.20%	74.10%	89.30%	42.50%	18
19	Jodhpur	Jaisalmer	9.10%	8.40%	24.00%	27.30%	54.60%	91.90%	35.90%	29
20	Jodhpur	Jalor	10.60%	15.40%	36.00%	36.90%	95.00%	95.00%	48.20%	11
21	Jodhpur	Jodhpur	14.30%	5.30%	24.10%	24.50%	86.00%	94.50%	41.50%	21
22	Jodhpur	Pali	38.50%	1.10%	0.60%	0.90%	95.00%	95.00%	38.50%	23
23	Jodhpur	Sirohi	51.50%	7.10%	13.80%	14.30%	86.70%	80.80%	42.40%	19
	AVG. COVERAGE OF JODHPUR ZONE		22.20%	9.50%	21.40%	22.70%	81.90%	91.10%	41.50%	
24	Kota	Baran	46.10%	4.70%	10.50%	10.20%	95.00%	88.50%	42.50%	17
25	Kota	Bundi	36.40%	9.00%	37.50%	50.40%	95.00%	95.00%	53.90%	7
26	Kota	Jhalawar	32.70%	5.10%	14.60%	18.30%	95.00%	95.00%	43.40%	15
27	Kota	Kota	30.00%	4.60%	20.20%	21.50%	95.00%	95.00%	44.40%	13
	AVG. COVERAGE OF KOTA ZONE		36.30%	5.80%	20.70%	25.10%	95.00%	93.40%	46.00%	
28	Udaipur	Banswara	70.40%	6.20%	10.90%	12.50%	95.00%	95.00%	48.30%	9
29	Udaipur	Chittaurgarh	58.10%	14.20%	48.50%	39.30%	95.00%	95.00%	58.30%	4
30	Udaipur	Dungarpur	28.80%	0.30%	1.20%	1.10%	95.00%	95.00%	36.90%	27
31	Udaipur	Pratapgarh	67.00%	0.00%	0.00%	0.00%	95.00%	95.00%	42.80%	16
32	Udaipur	Rajsamand	36.40%	20.20%	45.00%	53.40%	95.00%	95.00%	57.50%	5
33	Udaipur	Udaipur	17.50%	4.90%	14.00%	16.70%	95.00%	75.10%	37.20%	26
	AVG. COVERAGE OF UDAIPUR ZONE		46.40%	7.60%	19.90%	20.50%	95.00%	91.70%	46.90%	
	AVG. COVERAGE OF RAJASTHAN STATE		35.80%	7.32%	21.95%	22.70%	91.70%	92.10%	45.30%	

Table H: Outlier Districts List For Month of May-2022 (as on 27-June-2022)

S.No.	Outlier Districts (28)	Name of Outlier Indicator	Coverage Percentage as per distri	Changed to
1	Ajmer	Adoloscent (10-19)Years Out of school girls given IFA Blue tablets 4 time & above	176.40%	95.00%
2	Alwar	Same as above	465.40%	95.00%
3	Banswara	Same as above	133.40%	95.00%
4	Baran	Same as above	172.00%	95.00%
5	Bharatpur	Same as above	623.40%	95.00%
6	Bhilwara	Same as above	167.10%	95.00%
7	Bikaner	Same as above	166.40%	95.00%
8	Bundi	Same as above	162.00%	95.00%
9	Chittaurgarh	Same as above	529.10%	95.00%
10	Churu	Same as above	472.50%	95.00%
11	Dausa	Same as above	3407.90%	95.00%
12	Dhaulpur	Same as above	211.00%	95.00%
13	Dungarpur	Same as above	213.80%	95.00%
14	Ganganagar	Same as above	152.60%	95.00%
15	Hanumangarh	Same as above	118.40%	95.00%
16	Jaipur	Same as above	3661.70%	95.00%
17	Jalor	Same as above	242.20%	95.00%
18	Jhalawar	Same as above	361.40%	95.00%
19	Jhunjhunu	Same as above	3590.80%	95.00%
20	Kota	Same as above	289.00%	95.00%
21	Nagaur	Same as above	246.60%	95.00%
22	Pali	Same as above	112.10%	95.00%
23	Pratapgarh	Same as above	206.60%	95.00%
24	Rajsamand	Same as above	199.80%	95.00%
25	Sawai Madhopur	Same as above	325.00%	95.00%
26	Sikar	Same as above	612.50%	95.00%
27	Tonk	Same as above	452.10%	95.00%
28	Udaipur	Same as above	186.00%	95.00%

S.No.	Outlier Districts (23)	Name of Outlier Indicator	Coverage Percentage as per distri	Changed to
		Pregnant Women		
1	Banswara	Given 180 IFA tablets (incluc	97.00%	95.00%
2	Bharatpur	Same as above	144.50%	95.00%
3	Bhilwara	Same as above	106.80%	95.00%
4	Bikaner	Same as above	99.00%	95.00%
5	Bundi	Same as above	97.50%	95.00%
6	Chittaurgarh	Same as above	98.30%	95.00%
7	Churu	Same as above	95.10%	95.00%
8	Dausa	Same as above	113.20%	95.00%
9	Dhaulpur	Same as above	125.40%	95.00%
10	Dungarpur	Same as above	97.00%	95.00%
11	Ganganagar	Same as above	99.30%	95.00%
12	Hanumangarh	Same as above	151.20%	95.00%
13	Jalor	Same as above	95.50%	95.00%
14	Jhalawar	Same as above	95.20%	95.00%
15	Jhunjhunu	Same as above	146.80%	95.00%
16	Karauli	Same as above	124.80%	95.00%
17	Kota	Same as above	164.40%	95.00%
18	Pali	Same as above	98.60%	95.00%
19	Pratapgarh	Same as above	137.10%	95.00%
20	Rajsamand	Same as above	134.70%	95.00%
21	Sawai Madhopur	Same as above	100.30%	95.00%
22	Sikar	Same as above	126.00%	95.00%
23	Tonk	Same as above	131.10%	95.00%
